# Tolerability and Acceptance of Switching from Brand to Generic Glatiramer Acetate in Multiple Sclerosis

**DOI:** 10.3390/jcm13102780

**Published:** 2024-05-09

**Authors:** Isabella Maraffi, Giulia Mallucci, Giulio Disanto, Rosaria Sacco, Massimiliano Tiberti, Claudio Gobbi, Chiara Zecca

**Affiliations:** 1 Neurocenter of Southern Switzerland, Ente Ospedaliero Cantonale (EOC), 6900 Lugano, Switzerland; 2 Faculty of Biomedical Sciences, Università della Svizzera Italiana, 6900 Lugano, Switzerland

**Keywords:** multiple sclerosis, glariramer acetate, Copaxone

## Abstract

**Background:** The costs of disease-modifying therapies (DMTs) for multiple sclerosis (MS) have increased interest in generic alternatives. **Methods:** This prospective and observational study aims to investigate the safety, tolerability, and acceptance of switching from brand glatiramer acetate (GA) 40 mg/mL three times per week (Copaxone^®^) to generic GA 40 mg/mL three times per week (Glatiramyl^®^). Conducted at the Neurocenter of Southern Switzerland from September 2020 to September 2021, the study enrolled 27 patients; 21 completed the study. Participants reported on local and systemic side effects three months before and after the switch, and on switch acceptance by means of visual analogue scales (from 0 to 10). **Results:** Results indicated that those on generic GA experienced fewer local (81.0% vs. 96.3%) and systemic (33.3% vs. 59.3%) adverse events than with the brand drug. The median intensity of local adverse events was 8 (4–20) on generic GA vs. 16 (9–22) on brand GA, while the median intensity of systemic adverse events was similar between generic and brand GA [0 (0–27) vs. 0 (0–21.5), respectively]. Seventy-one percent of participants rated their acceptance of generic GA as 7/10 or higher. **Conclusions:** The results suggest that switching from brand to generic GA 40 mg/mL is safe, well-tolerated, and accepted by patients with MS.

## 1. Introduction

Medications represent a significant and escalating component of the high costs associated with multiple sclerosis (MS) care [1]. Over the past two decades, the cost of disease-modifying therapies (DMTs) for MS has seen a dramatic rise. From 2000 to 2013, the prices for DMTs surged by four to five times, with the average annual cost for most DMTs now exceeding $70,000 [2,3]. In Switzerland, data from 2011 to 2015 reveal that the mean annual cost of basic health insurance for medications was notably higher for individuals with MS compared to those without (28,000 vs. 4500 CHF, respectively) [4].

The expiration of patents for the first wave of MS treatments has opened the door for the development of generic versions.

The U.S. Food and Drug Administration (FDA) granted approval to a generic version of glatiramer acetate (GA) 20 mg/mL in 2015 based on the demonstration of pharmaceutical equivalence [5]. However, this process does not typically address the potential for clinical outcome differences or the identification of adverse events that may be more common with generic drugs. Furthermore, generic drugs may differ in their inactive ingredients from the brand-name versions with potential differences in safety or efficacy.

In contrast, the European Medicines Agency (EMA) classified GA as a complex non-biological product necessitating the inclusion of clinical trial data for marketing authorization application. In this context, the GATE trial conducted over a 9-month period in patients with relapsing-remitting MS (RRMS) demonstrated that the generic GA 20 mg/mL was equivalent in efficacy, safety, and tolerability to the brand GA 20 mg/mL (Copaxone^®^, Teva) [6]. Additionally, the extension phase of this trial revealed that switching patients from brand to generic GA could be achieved without compromising efficacy, safety, or tolerability [7].

Subsequently, a new 40 mg/mL dosage of brand GA was approved and administered three times per week [8]. When comparing the qualitative features of the brand and generic GA (both 20 and 40 mg/mL), the sole difference lies in the increased concentration of the 40 mg/mL GA formulation. Consequently, EMA and FDA assumed equivalence of efficacy, safety, and tolerability between generic and brand GA 40 mg/mL, as doubling the concentration of GA does not alter any of the drug substance’s characteristics.

Despite the regulatory advancements and approvals, significant research gaps remain, particularly regarding the safety, tolerability, and patient acceptance of switching from brand-name to generic GA 40 mg/mL. Such a switch may not only introduce clinical uncertainties but could also significantly impact patients psychologically. Concerns about generic substitution are particularly tricky in the treatment of chronic diseases like MS, where patients may be sensitive to changes in medication regimen. Our study aims to contribute to fill this research gap by focusing on safety, tolerability, and acceptance of generic GA 40 mg/mL (Glatiramyl^®^) among MS patients who have switched from the brand GA 40 mg/mL (Copaxone^®^). By addressing these concerns, the study seeks to provide insights into the broader implications of switching to generic medications within this patient population, encompassing both medical outcomes and patient perspectives.

## 2. Methods

### 2.1. Setting and Study Design

This was a prospective, observational, monocentric study, conducted at the third-level Multiple Sclerosis Center at the Neurocenter of Southern Switzerland in Lugano, Switzerland, spanning from September 2020 to September 2021. This study was conducted in accordance with the Declaration of Helsinki, and approval was obtained from the Ethics Committee (CE2454).

**Figure 1:**
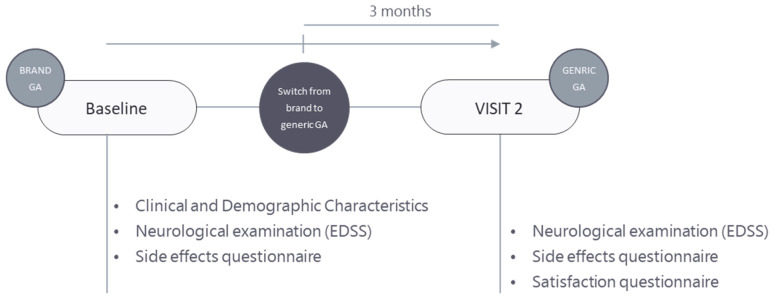
Study design. At baseline (T0), patients are treated with brand GA, demographic and clinical data are collected, a neurological examination including Neurostatus EDSS is performed, and patients complete the Side Effects Questionnaire. Following the switch to generic GA (T switch), patients are reassessed 3 months later at visit 2 (T2), where they undergo another neurological examination, fill in the Side Effects Questionnaire, and evaluate their satisfaction with the generic GA using the Satisfaction Questionnaire.

Figure 1 depicts the layout of our study. Patients were prospectively enrolled. At baseline (T0), enrolled patients underwent a comprehensive neurological examination and completed the Side Effects Questionnaire, which tracked both local and systemic side effects they had experienced over the previous three months while using brand GA. During this baseline visit, neurologists also collected essential clinical and demographic data including age, sex, onset of MS, course of MS, duration of MS, Expanded Disability Status Scale (EDSS), and duration of treatment with brand GA. Visit 2 (T2) took place three months after the patients had switched from branded to generic GA, a change made in accordance with clinical practice guidelines (noted as T switch). In this follow-up visit, patients again underwent a neurological examination, in line with clinical practice, and completed the Side Effect Questionnaire to note any new or ongoing side effects since the medication switch. Additionally, patients evaluated their satisfaction with the switch using the Satisfaction Questionnaire.

### 2.2. Participants and Inclusion Criteria

All patient consented to participate in the study and provided written consent to the use of their clinical data. The inclusion criteria comprised the following: (1) a diagnosis of RRMS or clinically isolated syndrome (CIS) according to riveted MS criteria 2017 [9]; (2) current treatment with brand GA 40 mg/mL three times per week, according to Swiss label; (3) planning to switch according to clinical practice to generic GA 40 mg/mL three times per week; (4) a minimum of 12 months of clinical and radiological stability, characterized by the absence of relapses, no increase in the EDSS score, and no contrast-enhancing lesions or new/enlarged T2 lesions at MRI, according to local clinical practice. Patients who were not sufficiently fluent in Italian (protocol language) to understand the study procedures and provide informed consent were excluded from the study.

### 2.3. Clinical Assessments

All participants underwent a standard neurological examination, performed by a senior neurologist certified to perform Neurostatus EDSS.

### 2.4. Side Effects Questionnaire

To assess the side effects associated with GA treatment experienced by patients over the previous three months, we used a detailed Side Effects Questionnaire. This tool captured local side effects at the injection site, including pain, swelling, itching, redness, lipoatrophy, hematoma, induration, necrosis, and numbness relative to the injection site. Systemic side effects such as flushing, chest pain, palpitations, anxiety, dyspnoea, neck constriction, urticaria, gastrointestinal symptoms, shivers, fever, and headache were also recorded. Participants could seek clarification from the treating neurologist regarding any side effects they did not understand. The intensity of each side effect was evaluated using a Visual Analog Scale (VAS) ranging from 0 (indicating no adverse event) to 10 (indicating maximum intensity of the adverse event). They were also asked to report the onset time (within 2, 15, or 120 min post-injection), duration (less than 30 min, 30 min to 24 h, 1 to 7 days, or more than 7 days), and frequency (as a percentage of total injections) of each side effect. The Side Effects Questionnaire was administered at Baseline and at Visit 2. To reduce recall bias, we implemented a retrograde questioning technique, asking patients to report their experiences from the most recent back to the more distant. This method, coupled with the use of clear and specific language in our questionnaires and comprehensive evaluations to confirm that patients were not psychologically or physically impaired, helped to clarify the relevant time periods and improve the precision of the data collected [10].

### 2.5. Satisfaction Questionnaire

To evaluate patient satisfaction with the switch to generic GA, a satisfaction questionnaire was administered at Visit 2. Patients evaluated their acceptance of the generic GA using a VAS, which ranged from 0 (no acceptance) to 10 (excellent acceptance).

### 2.6. Statistical Analysis

Descriptive statistics were employed to summarize the findings; for continuous variables, we calculated means and medians along with standard deviations and interquartile ranges (IQR). For categorical variables, frequencies were calculated. Statistical tests were not conducted due to the limited sample size, which restricted our ability to conduct statistically meaningful tests.

## 3. Results

A total of 27 participants treated with brand GA 40 mg/mL three times per week were included in the study. Demographic and clinical characteristics are presented in Table 1. Median age was 48 (38.5–58.0) years; most patients were female (74.1%) and had RR MS (77.8%). Patients had been treated with brand GA for a median of 84.0 months (IRQ 33–119.5). Six of the included patients did not proceed with the planned switch to generic GA at a dosage of 40 mg/mL three times a week for various reasons. Specifically, two patients experienced a worsening of their disability that necessitated an escalation in their treatment plan, three patients expressed a preference to continue with their current brand GA treatment, and one patient opted for a switch to an oral treatment alternative. Consequently, the follow-up questionnaire was administered to the remaining 21 patients who had completed 3 months of treatment with the generic GA.

### 3.1. Local Side Effects

The proportion of patients who did not experience local side effects was higher for generic GA (19.0%) than brand GA (3.7%). The median number of local side effects per patient was 2 (1–5) and 4 (2–6) for generic GA and brand GA, respectively. The proportion of patients with ≥3 local side effects was 47.6% for generic GA and 70.4% for brand GA. The most frequently reported side effects were pain and redness for generic GA, and pain and swelling for brand GA. Most patients experienced local side effects within 2 min of injection (88.2% for generic GA vs. 84.6% for brand GA) (Table 2). In order to estimate an overall intensity local symptom score, we summed the VAS scores across all local symptoms within each individual patient. This median overall intensity score was 8 (4–20) for generic GA vs. 16 (9–22) for brand GA. When present, most local side effects lasted for more than 1 week for both generic GA (47.1%) and brand GA (46.2%), and they occurred with a similar frequency between generic GA (50% [33–100%]) and brand GA (50% [33–100%]).

### 3.2. Systemic Side Effects

The proportion of patients who did not experience systemic side effects was higher for generic GA (66.7%) than brand GA (40.7%). The median number of systemic side effects per patient was 0 (0–1) and 1 (0–4) for generic GA and brand GA, respectively. The proportion of patients with ≥3 systemic side effects was 19% for generic GA and 33.3% for brand GA. The most frequently reported side effects were flushing, palpitations, and shivering for both generic and brand GA (Table 3). As with local side effects, most patients experienced systemic side effects within 2 min of injection (71.4% for generic GA vs. 62.5% for brand GA). The median overall intensity systemic symptom score was 0 (0–27) for generic GA vs. 0 (0–21.5) for brand GA. When present, most systemic side effects lasted for more than 30 min but less than 24 h for both generic GA (71.4%) and brand GA (56.3%). The median frequency of occurrence was similar between generic GA (33% [10–50%]) and brand GA (33% [10–50%]).

### 3.3. Treatment Acceptance

Three months after switching treatments, the median (IRQ) treatment acceptance was 7 (6.25–9). Furthermore, 71% of patients (15 out of 21) rated their acceptance of the new treatment as 7 out of 10 or higher.

## 4. Discussion

Our study aimed to investigate the safety, tolerability, and acceptance of generic GA (Glatiramyl^®^) at a dosage of 40 mg/mL, administered three times per week, among patients with MS switching from its brand version (Copaxone^®^). This investigation is particularly pertinent in the context of escalating healthcare costs associated with MS care and the potential for generic drugs to provide more affordable treatment alternatives without sacrificing efficacy and safety.

Distinct from previous studies that mainly examined lower dosages [6,7,8], our research investigated the switch from brand to generic GA 40 mg/mL, three times a week. Local and systemic tolerability is crucial when it comes to injectable DMTs for MS, as it significantly impacts treatment satisfaction and adherence due to side effect burden [11]. Indeed, non-adherence is associated with increased risk of relapses and higher healthcare utilization. Our study addresses this knowledge gap.

Notably, our data suggest that patients switching from brand to generic GA 40 mg/mL three times a week displayed more favorable tolerability of treatment during the first 3 months of therapy when compared to the last 3 months on brand GA 40 mg/mL three times per week. This is evidenced by a notable improvement within the same cohort of patients, as a higher proportion reported no local side effects when using generic GA (19.0%) compared to brand GA (3.7%). Furthermore, the median number of local side effects was lower for patients on generic GA. These observations are supported by the lower reported median overall intensity scores for local symptoms with generic GA. Similarly, we observed a lower number of systemic side effects in patients after switching to generic GA. A higher proportion of these patients reported no systemic side effects with the generic vs. brand GA formulation. The types of systemic side effects remained consistent across both treatments, indicating that switching to the generic version did not introduce any new adverse events. Despite the small sample size, our within-subject comparison of drug tolerability highlights the potential of generic GA as a well-tolerated alternative to brand GA, thus contributing to reassure healthcare providers and patients. A direct comparison of our data with those from the switch from brand to generic GA at 20 mg/mL, as presented in the GATE extension study, is limited both by the different drug dosage, concentration, and posology, as well as by the different statistical approaches, i.e., between-group vs. intra-individual comparison [7].

The acceptance of generic GA among our study participants was notably high, as evidenced by the median acceptance score of 7/10. This positive reception is crucial for the successful switch to generic formulations, given that patient perceptions can profoundly impact treatment adherence and outcomes. It is important to note that this finding stands in contrast to the literature from neurology, especially in the antiepileptic field, and other disciplines, where generics are often associated with distress and worse outcomes [12]. Negative perceptions of generics can lead to an increase in the nocebo effect following the switch from a branded to a generic alternative, with greater complaints of side effects and beliefs that the new medication is less effective [13]. Ultimately, this may lead to a significant number of patients switching back to the branded medication, thereby reducing the cost savings for the health system. This underscores the complexity of patient acceptance and the potential influence of specific drug contexts and disease areas on perceptions of generic medications.

Despite the mentioned promising results, this study is limited by factors that could influence its robustness and applicability. The assessments of brand and generic GA via the Side Effects Questionnaire usage could introduce recall bias, potentially skewing reports of side effects and satisfaction. However, we believe this impact is mitigated by the relatively short three-month recall period, which is less likely to suffer from the inaccuracies often seen with longer durations. Additionally, to further minimize potential recall bias, we employed a retrograde questioning technique. This approach, combined with the use of precise language in our questionnaires and thorough assessments to ensure patients were not psychologically or physically impaired, helped to clarify the timeframe in question and enhance the accuracy of the data collected [10]. Additionally, the small sample size, the short duration, and the study’s monocentric design limit the generalizability of our findings. Thus, the outcomes observed may not be representative of broader populations, as drug tolerability and acceptance can be influenced by genetic and cultural factors [14]. Future research should aim to include a larger, more diverse cohort, and possibly expand to a multicentric format. This would enhance the statistical power and relevance of the data across different populations. Finally, employing blinded, prospective study designs could help mitigate some of the current limitations by providing more controlled and direct observations of the effects of switching to generic GA.

Despite these constraints, our research adds to the accumulating evidence supporting the safety and tolerability of generic GA, while additionally providing important insights into the very good acceptance levels of generic medications in MS patients.

## 5. Conclusions

This study assessed the safety, tolerability, and acceptance of switching from brand-name to generic GA 40 mg/mL in MS patients. Our findings highlight that generic GA is a cost-effective, well-tolerated alternative, with fewer reported side effects and high acceptance rates. This suggests a favorable impact of generic GA on treatment adherence and patient satisfaction. However, limitations such as the study’s small sample size and monocentric design could impact the generalizability of these results. Future research should focus on larger, multicentric, prospective studies to confirm these findings and investigate the long-term effects of switching to generic GA.

## Figures and Tables

**Table 1:** Demographic and Clinical Characteristics of Study Participants. Abbreviations: CIS—clinically isolated syndrome; EDSS—expanded disability status scale; GA—glatiramer acetate; IQR—interquartile range; RRMS—relapsing remitting multiple sclerosis.

Variable	Median (IQR)/Count (%)
Age, year (IQR)	48 (38.5–58.0)
Sex	
*Female, n (%)*	20 (74.1)
*Male, n (%)*	7 (25.9)
Disease course	
*CIS, n (%)*	3 (11.1)
*RRMS, n (%)*	24 (77.8)
EDSS	2.0 (1.75–2.5)
Disease duration, months (IQR)	120 (54–192)
Duration of treatment with brand GA, months (IQR)	84 (33–119.5)

**Table 2:** Summary statistics for experienced local side effects during treatment with generic vs. brand GA. Abbreviations: GA—glatiramer acetate; IQR—interquartile range.

Local Side Effects		Generic GA		Brand GA	
		n/Median	%/IQR	n/Median	%/IQR
Patients without side effects		4	19.0	1	3.7
Number of side effects per patient		2	1–5	4	2–6
Sum of overall VAS scores		8	4.0–20.0	16	9–22.5
Number of patients with each side effect	Pain	11	52.4	18	66.7
	Swelling	9	42.9	18	66.7
	Itching	7	33.3	10	37.0
	Redness	11	52.4	17	63.0
	Lipoatrophy	3	14.3	8	29.6
	Haematoma	8	38.1	14	51.9
	Induration	8	38.1	17	63.0
	Necrosis	0	0.0	0	0.0
	Numbness	4	19.0	5	18.5
Minimum time from injection to side effect per patient	<2 min	15	88.2	22	84.6
	2–15 min	1	5.9	2	7.7
	15–120 min	0	0.0	1	3.8
	>120 min	1	5.9	1	3.8
Maximum duration of side effect per patient	<30 min	3	17.6	2	7.7
	30 min–24 h	2	11.8	5	19.2
	24 h–1 sett	4	23.5	7	26.9
	>1 sett	8	47.1	12	46.2

**Table 3:** Summary statistics for experienced systemic side effects during treatment with generic vs. brand GA. Abbreviations: GA—glatiramer acetate; IQR—interquartile range.

Systemic Side Effects		Generic GA		Brand GA	
		n/Median	%/IQR	n/Median	%/IQR
Patients without side effects		11	40.7	14	66.7
Number of side effects per patient		0	0–1	0	0–4
Sum of overall VAS scores		0	0–27	0	0–21.5
Number of patients with each side effect	Flushing	4	19.0	8	29.6
	Chest pain	1	4.8	5	18.5
	Palpitations	3	14.3	9	33.3
	Anxiety	2	9.5	6	22.2
	Dyspnea	0	0.0	6	22.2
	Neck costr	2	9.5	3	11.1
	Urticaria	1	4.8	2	7.4
	GI	1	4.8	1	3.7
	Shivers	3	14.3	6	22.2
	Fever	0	0.0	2	7.4
	Headache	0	0.0	2	7.4
Minimum time from injection to side effect per patient	<2 min	5	71.4	10	62.5
	2–15 min	2	28.6	3	18.8
	15–120 min	0	0.0	3	18.8
	>120 min	0	0.0	0	0.0
Maximum duration of side effect per patient	<30 min	2	28.6	5	31.3
	30 min–24 h	5	71.4	9	56.3
	24 h–1 sett	0	0.0	1	6.3
	>1 sett	0	0.0	1	6.3

## Data Availability

The data presented in this study are available on reasonable request from the corresponding author due to privacy concerns related to patient data (specify the reason for the restriction).
